# Lysophosphatidylcholine Acyltransferase 3 Is the Key Enzyme for Incorporating Arachidonic Acid into Glycerophospholipids during Adipocyte Differentiation

**DOI:** 10.3390/ijms131216267

**Published:** 2012-12-03

**Authors:** Miki Eto, Hideo Shindou, Andreas Koeberle, Takeshi Harayama, Keisuke Yanagida, Takao Shimizu

**Affiliations:** 1 National Center for Global Health and Medicine, 1-21-1 Toyama, Shinjuku-ku, Tokyo 162-8655, Japan; E-Mails: metou-tky@umin.ac.jp (M.E.); harayama-t@umin.net (T.H.); tshimizu@m.u-tokyo.ac.jp (T.S.); 2 Department of Biochemistry and Molecular Biology, Faculty of Medicine, The University of Tokyo, 7-3-1 Hongo, Bunkyo-ku, Tokyo 113-0033, Japan; E-Mails: andreas.koeberle@uni-jena.de (A.K.); yanagida-tky@umin.ac.jp (K.Y.); 3 Chair of Pharmaceutical/Medicinal Chemistry, Institute of Pharmacy, Friedrich-Schiller-University, Jena 07743, Germany

**Keywords:** glycerophospholipid, lysophospholipid acyltransferase, adipocyte, C3H10T1/2, LPCAT3, Lands’ cycle, arachidonic acid

## Abstract

Cellular membranes contain glycerophospholipids, which have important structural and functional roles in cells. Glycerophospholipids are first formed in the *de novo* pathway (Kennedy pathway) and are matured in the remodeling pathway (Lands’ cycle). Recently, lysophospholipid acyltransferases functioning in Lands’ cycle were identified and characterized. Several enzymes involved in glycerophospholipid biosynthesis have been reported to have important roles in adipocytes. However, the role of Lands’ cycle in adipogenesis has not yet been reported. Using C3H10T1/2, a cell line capable of differentiating to adipocyte-like cells *in vitro*, changes of lysophospholipid acyltransferase activities were investigated. Lysophosphatidylcholine acyltransferase (LPCAT), lysophosphatidylethanolamine acyltransferase (LPEAT) and lysophosphatidylserine acyltransferase (LPSAT) activities were enhanced, especially with 18:2-CoA and 20:4-CoA as donors. Correspondingly, mRNA expression of LPCAT3, which possesses LPCAT, LPEAT and LPSAT activities with high specificity for 18:2- and 20:4-CoA, was upregulated during adipogenesis. Analysis of acyl-chain compositions of phosphatidylcholine (PC), phosphatidylethanolamine (PE) and phosphatidylserine (PS) showed a change in their profiles between preadipocytes and adipocytes, including an increase in the percentage of arachidonic acid-containing phospholipids. These changes are consistent with the activities of LPCAT3. Therefore, it is possible that enhanced phospholipid remodeling by LPCAT3 may be associated with adipocyte differentiation.

## 1. Introduction

Glycerophospholipids are structural and functional components of cellular membranes, as well as precursors of a variety of lipid mediators. There are different classes of glycerophospholipids, such as phosphatidic acid (PA), phosphatidylcholine (PC), phosphatidylethanolamine (PE), phosphatidylglycerol (PG), phosphatidylinositol (PI), phosphatidylserine (PS) and cardiolipin (CL), which contain distinct compositions of fatty acids in different cell types and tissues [1–4]. Glycerophospholipids are initially generated and then matured in two distinct pathways. In the *de novo* pathway (Kennedy pathway), glycerophospholipids are formed from glycerol-3-phosphate [5]; the key enzymes functioning in the *de novo* pathways have been characterized [6,7]. In the remodeling pathway (termed Lands’ cycle), the concerted actions of phospholipase A_2_s (PLA_2_s) and lysophospholipid acyltransferases (LPLATs) function to establish the asymmetry and high diversity of glycerophospholipids [8–11]. Recently, several LPLATs functioning in the Lands’ cycle have been cloned and characterized by various laboratories, including ours [12,13]. These enzymes were found from the 1-acylglycerol-3-phosphate *O*-acyltransferase (AGPAT) family and the membrane-bound *O*-acyltransferase (MBOAT) family, each with distinct motifs essential for their activities [14–17]. Lysophosphatidylcholine acyltransferase (LPCAT) 3 is a member of the MBOAT family and exhibits LPCAT, lysophosphatidylethanolamine acyltransferase (LPEAT) and lysophosphatidylserine acyltransferase (LPSAT) activities, with preference for 18:2- and 20:4-CoA [18–21].

Adipose tissues are not only places for fat storage, but are also important endocrine organs [22]. Research on adipose tissue development and adipocyte differentiation is therefore important. Several enzymes involved in glycerophospholipid biosynthesis have been reported to have important functions in adipocytes. Lysophosphatidic acid acyltransferase 2 (LPAAT2), an enzyme functioning in the *de novo* pathway, is known to have an important role in adipogenesis [23,24]; mutations in this gene cause Berardinelli-Seip congenital lipodystrophy [25]. CTP: phosphocholine cytidylyltransferase, an enzyme involved in the biosynthesis of PC in the *de novo* pathway, is reported to be important for lipid droplet expansion [26]. Phosphatidylethanolamine *N*-methyltransferase, which synthesizes PC from PE, was also reported to have roles in adipogenesis [27]. However, the role of the Lands’ cycle in adipocytes has not yet been reported.

C3H10T1/2 [28] is a mesenchymal stem cell line that is capable of differentiating into adipocytes [29]. Using C3H10T1/2 cells, we investigated the changes of mRNA expression of LPLATs and changes of phospholipid composition that occur during adipogenesis. We found LPCAT3 was selectively upregulated during adipogenesis, accompanied by changes in cellular phospholipid compositions consistent with the known activities of LPCAT3. Our results show a possibility that LPCAT3-mediated enhanced phospholipid remodeling may be associated with adipocyte differentiation.

## 2. Results and Discussion

### 2.1. Lysophospholipid Acyltransferase Activities Were Increased during Adipogenesis

To examine the role of the Lands’ cycle in adipogenesis, C3H10T1/2 cells were differentiated into adipocytes. In this study, undifferentiated C3H10T1/2 cells are termed preadipocytes. LPCAT, LPEAT and LPSAT activities of day 0 preadipocytes and day 8 adipocytes were measured with 16:0-, 18:1-, 18:2-, 20:4- and 22:6-CoA as donors. LPCAT, LPEAT and LPSAT activities were increased with all acyl-CoAs, especially with 18:2-CoA and 20:4-CoA (Table 1). These data suggest that the LPLAT activities in the Lands’ cycle enhance with adipocyte differentiation.

### 2.2. LPCAT3 mRNA Was Increased during Adipocyte Differentiation

**Figure 1:**
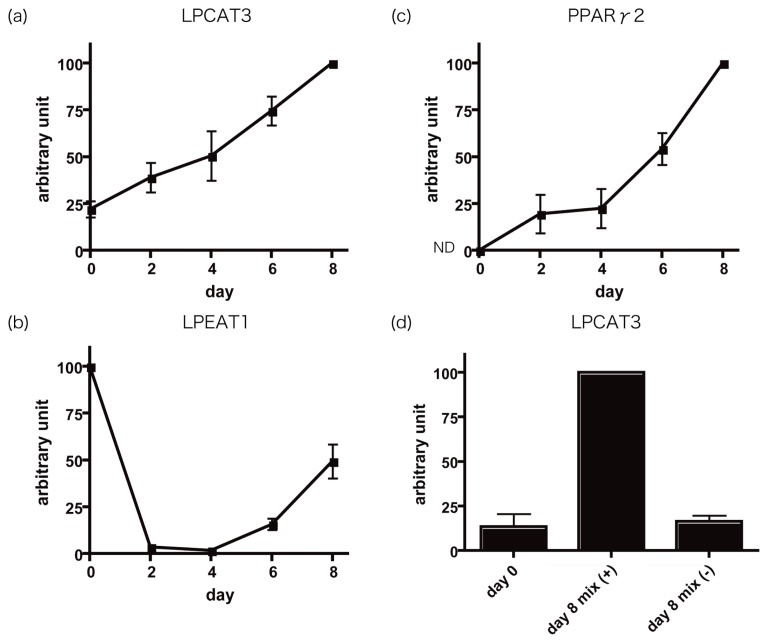
mRNA expression of LPCAT3 (**a**), LPEAT1 (**b**) and PPARγ2 (**c**) during adipocyte differentiation. mRNA expression of LPCAT3 in day 0 cells and day 8 cells, with or without induction (**d**). mix (+) indicates cells cultured with the induction mixture, and mix (−) indicates cells cultured without the induction mixture. The data shown are values normalized by 36B4, a housekeeping gene. The arbitrary units were calculated as setting the maximum value as 100. The figures show the mean ± SE of three independent experiments. ND, not detected.

To investigate the mechanism of the increase in LPLAT activities, mRNA expression of known enzymes, which posses LPCAT, LPEAT or LPSAT activities, were determined in C3H10T1/2 cells during adipocyte differentiation. Using quantitative PCR analysis, expression levels of LPCAT1, LPCAT2, LPCAT3, LPCAT4 and LPEAT1 were examined. The values were normalized by mRNA level of 36B4, a housekeeping gene. mRNA expression of LPCAT3 (Figure 1a), which has LPCAT, LPEAT, and LPSAT activities with high substrate specificities for 18:2-CoA and 20:4-CoA, was upregulated during differentiation. LPCAT3 mRNA was not enhanced when the induction mixture was not added to the medium (Figure 1d), showing that the effect on LPCAT3 expression was not caused by the confluency of the cells. LPEAT1 mRNA was decreased dramatically on day 2 and was gradually increased again (Figure 1b). LPCAT1, LPCAT2 and LPCAT4 mRNA were not detected (data not shown). mRNA expression of peroxisome proliferator-activated receptor γ 2 (PPARγ2), a marker for adipocyte differentiation, was not detected on day 0 and was gradually increased during differentiation (Figure 1c), which shows that the cells actually differentiated into adipocytes.

Our data suggest increased LPCAT3 expression leads to enhanced LPCAT, LPEAT and LPSAT activities important for adipocyte differentiation. The decreased expression of LPEAT1 with high substrate specificity for 18:1-CoA also supports that acyltransferase activities utilizing 18:2-CoA and 20:4-CoA rather than 18:1-CoA may play important roles in adipocyte differentiation.

### 2.3. Change in Phospholipid Composition during Adipocyte Differentiation

To investigate whether the shift in LPLAT activities affect the fatty acid composition of phospholipids, PC, PE and PS species were compared between preadipocytes and adipocytes (Table 2). Lipids from day 0 preadipocytes and day 8 adipocytes were extracted and analyzed by liquid chromatography tandem mass spectrometry (LC-MS/MS). The signal intensities for each class of phospholipid were summed and the abundance of each species was calculated as the percentage of the sum.

The phospholipid profiles showed many changes between preadipocytes and adipocytes, especially for PC and PE. We observed that the percentage of phospholipid species probably containing arachidonic acid, such as 36:4 PC, 38:4 PC and 36:4 PE, was increased during differentiation. This might have been caused by the activity of LPCAT3. Increases in 32:1 PC and PE might have been caused by a different enzyme, such as stearoyl-CoA desaturase, which is known to be induced during differentiation [30].

To confirm that species that were increased (36:4 PC, 38:4 PC and 36:4 PE) actually contain arachidonic acid, we determined fatty acid species of PC and PE (Table 3). The signal intensities for each species were summed up and the percentage was calculated. The percentage of 16:0/20:4 PC, 18:0/20:4 PC and 16:0/20:4 PE were enhanced, which shows that arachidonic acid-containing species are increased during adipocyte differentiation.

### 2.4. Possible Role of LPCAT3 in Adipocyte Differentiation

In this study, we found that LPCAT3 mRNA is upregulated during differentiation of adipocytes. This enzyme exhibits LPCAT, LPEAT and LPSAT activities with 18:2-CoA and 20:4-CoA. We also discovered that LPCAT, LPEAT and LPSAT activities are enhanced during adipocyte differentiation, especially with 18:2-CoA and 20:4-CoA, thus correlating with the activities of LPCAT3. Furthermore, the fatty acid compositions of phospholipids changed during adipocyte differentiation, including an increase in arachidonic acid-containing species. Taken together, these data suggest that induction of LPCAT3 expression during adipocyte differentiation leads to enhanced LPCAT, LPEAT and LPSAT activities and increased incorporation of arachidonic acid into membrane phospholipids.

**Figure 2:**
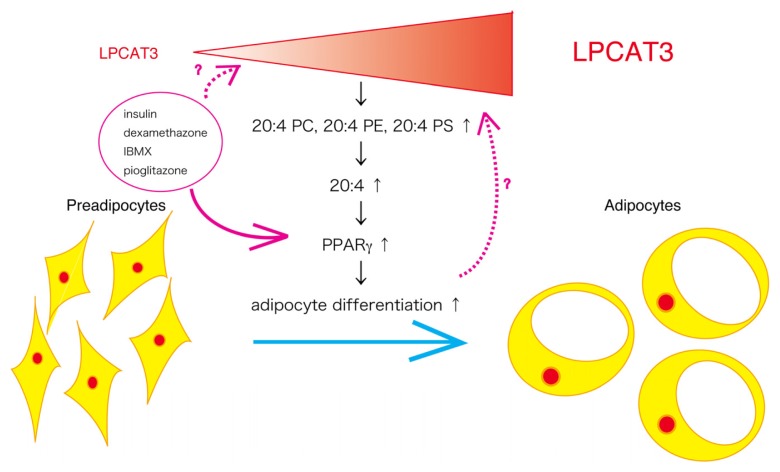
A proposed scheme for the role of LPCAT3 in adipocyte differentiation. LPCAT3 mRNA expression is induced during adipocyte differentiation, leading to increased PC, PE and PS containing arachidonic acid. Abundant arachidonic acid in membrane phospholipids enhances activity of PPARγ by producing endogenous ligands, thereby promoting adipocyte differentiation.

Arachidonic acid-containing phospholipids are important for synthesizing eicosanoids [10]. Some of these mediators, such as 15-deoxy-Δ^12,14^-prostaglandin J2, are known to act as endogenous ligands for PPARγ [31]. LPCAT3-mediated arachidonic acid incorporation into membrane phospholipids may promote production of endogenous lipid ligands for PPARγ important for adipogenesis and adipocyte function. Arachidonic acid is also known to promote differentiation of preadipocytes and adipose tissue development through prostacyclin signaling [32]. There are several clinical reports suggesting a role of arachidonic acid content of glycerophospholipids for adipocyte function. One report shows a positive correlation between BMI and adipose tissue glycerophospholipids containing arachidonic acid in children [33]. Another report shows that high content of arachidonic acid in adipose tissue has an increasing risk of metabolic syndrome in adults [34]. From these reports, we can speculate that incorporation of arachidonic acid into membrane glycerophospholipids is an important step leading to activation of PPARγ and enhancing adipocyte differentiation. The results of our study show a possibility that LPCAT3 might have a role for maintaining membrane phospholipids rich in arachidonic acid and, thereby, leading to activation of PPARγ (Figure 2).

Although the changes we measured in LPCAT3 mRNA levels, acyltransferase activities and phospholipid compositions were associated with adipocyte differentiation, additional studies will be required to determine the roles of LPCAT3 and membrane phospholipid remodeling in preadipocytes and adipocytes *in vivo*.

In conclusion, this study revealed a novel possibility that the regulation of the phospholipid remodeling pathway by LPCAT3 is associated with adipocyte differentiation.

## 3. Experimental Section

### 3.1. Materials

Fetal bovine serum was purchased from Invitrogen (Carlsbad, CA, USA), 3-isobutyl-1-methylxanthine, dexamethazone, insulin and pioglitazone were purchased from Sigma (St. Louis, MO, USA). 14:0/14:0 PC and 14:0/14:0 PE standards were purchased from NOF Corporation (Tokyo, Japan). 17:0/20:4 PS standard, all lysophospholipids and all acyl-CoAs were purchased from Avanti Polar Lipids (Alabaster, AL, USA); 17:0–20:4 PS (Product number; LM-1302), 16:0 D31 Lyso PC (860397), 16:0 Lyso PE (856705), 16:0 Lyso PS (858142), 16:0 Coenzyme A (870716), 18:1 Coenzyme A (870719), 18:2 Coenzyme A (870736), 20:4 Coenzyme A (870721) and 22:6 Coenzyme A (870728). All organic solvents (methanol, chloroform and acetonitrile) used in this study are LC-MS grade, which were purchased from Wako (Osaka, Japan).

### 3.2. Cell Culture

C3H10T1/2 cells (ATCC) were grown in Dulbecco’s modified Eagle’s medium (DMEM) with 10% fetal bovine serum at 37 °C. Cells were grown to confluence (day 0). On day 0, they were induced to differentiate by changing the medium to DMEM with 10% fetal bovine serum, 0.5 mM 3-isobutyl-1-methylxanthine, 1 μM dexamethasone, 2.5 μM pioglitazone and 10 μg/mL insulin. On day 2, the medium was replaced with DMEM with 10% fetal bovine serum containing the same mixture. On day 4, the medium was changed to DMEM with 10% fetal bovine serum and 10 μg/mL insulin. From day 6, the medium was changed back to DMEM with 10% fetal bovine serum.

### 3.3. Quantitative RT-PCR Analysis

Total RNA was extracted from C3H10T1/2 cells on day 0, 2, 4, 6 and 8 using the RNeasy Lipid tissue Mini Kit (Qiagen GmbH, Hilden, Germany), and first-strand cDNA was subsequently synthesized using Superscript III (Invitrogen). PCRs (LightCycler System; Roche Applied Science, Mannheim, Germany) were performed using FastStart DNA Master SYBR Green I (Roche Applied Science). The mRNA levels were normalized to the 36B4, a housekeeping gene. The primers used are listed in Table 4.

### 3.4. Microsomal Protein Preparation and Lipid Extraction

C3H10T1/2 preadipocytes (day 0) and adipocytes (day 8) from 10 cm dishes were scraped into 1 mL of ice-cold buffer containing 20 mM Tris-HCl (pH 7.4), 300 mM sucrose and Complete Protease Inhibitor Cocktail (Roche Applied Science). Cells were sonicated three times on ice for 30 s using a probe sonicator (10 watts). After centrifugation for 10 min at 800× *g*, the supernatant was collected and centrifuged at 100,000× *g* for 1 h. The resultant pellets were resuspended in buffer containing 20 mM Tris-HCl (pH 7.4), 300 mM sucrose and 1 mM EDTA. Protein concentration was measured using a Bradford protein assay reagent (Bio-Rad, Hercules, CA, USA) and BSA (fraction V, fatty acid-free; Sigma) as a standard. For lipid analysis, 2 μg microsomal protein extracted, as mentioned before, from day 0 and day 8 C3H10T1/2 cells were dissolved in 200 μL methanol, centrifuged at maximum speed for 5 min and analyzed by liquid chromatography and tandem mass spectrometry (LC-MS/MS).

### 3.5. LPLAT Assays

The acyltransferase activity was measured according to Hishikawa *et al.*[18], Koeberle *et al.*[35] and Gijon *et al.*[20]. 0.5 μg protein was added to reaction mixtures containing multiple acyl-CoAs (5 μM each of 16:0-, 18:1-, 18:2-, 20:4-, and 22:6-CoA), multiple lysophospholipids (25 μM each of 16:0 d31 lysophosphatidylcholine, 16:0 lysophosphatidylethanolamine, and 16:0 lysophosphatidylserine; all lysophospholipids contain fatty acids at the *sn*-1 position), 100 mM Tris-HCl (pH 7.4) and 1 mM CaCl_2_, in a total volume of 0.1 mL. After incubation at 37 °C for 10 min, reactions were stopped by the addition of 0.3 mL of chloroform:methanol (1:2, *v*/*v*). Internal standards (14:0/14:0 PC, 14:0/14:0 PE and 17:0/20:4 PS) were added, total lipids were extracted by Bligh and Dyer method [36] and analyzed by LC-MS. For LPCAT activities, deuterium-labeled products (PC formed from d31 lysophosphatidylcholine and acyl-CoAs) were measured. For LPEAT and LPSAT activities, deuterium labeled lysophospholipids were not commercially available, so the products were calculated by subtracting endogenous lipids of the protein from the total. Endogenous lipids were measured by incubating the same protein (0.5 μg) in a reaction mixture that lacked acyl-CoA.

### 3.6. Reversed Phase Liquid Chromatography

Phospholipids were separated on an Acquity UPLC BEH C_8_ column (1.7 μm, 2.1 mm × 30 mm) using an Acquity Ultraperformance LC system (Waters, Milford, MA, USA). Flow rate was set at 0.8 mL/min, and column temperature was set at 45 °C. For LPLAT assays, PC and PE were separated by using a gradient of 25% 20 mM acqueous ammonium bicarbonate (A) and 75% acetonitrile (B) to A/B = 5/95 within 5 min, and PS was separated by using a linear gradient of A/B = 80/20 to A/B = 5/95 within 5 min. For analysis of phospholipid compositions, the lipids were separated by a gradient of A/B = 80/20 to A/B = 5/95 within 13.5 min.

### 3.7. Mass Spectrometry

The LC system was coupled to a TSQ Vantage Triple Stage Quadrupole Mass Spectrometer (Thermo Scientific, Waltham, MA, USA) with a HESI-II electrospray ionization source. The capillary temperature was heated to 280 °C, the vaporizer temperature to 550 °C, the sheath gas (nitrogen) pressure to 50 psi, the auxiliary gas (nitrogen) pressure to 15 psi and the collision gas (argon) pressure to 0.7 mtorr. The other parameters were set as recommended. Phospholipid species were identified by the headgroups and were analyzed with selected reaction monitoring of all the major species. PC and PE were analyzed by positive ion mode, and PS was analyzed by negative ion mode. PC was identified by the product ion of *m*/*z* = 184, PE was identified by the neutral loss of *m*/*z* = 141 and PS was identified by the neutral loss of *m*/*z* = 87. The collision energy was set at 35 eV for PC, 20 eV for PE and 20 eV for PS. Fatty acid composition of PC and PE were determined by detecting fatty acid anions in the negative mode, using selected reaction monitoring (collision energy 45 eV). Data show the intensities divided by the intensity of the internal standard (for LPLAT activities) or the percentage of the sum of all species detected (phospholipid composition).

### 3.8. Statistics

Statistical evaluations were performed by using Student’s *t*-test. Calculations were performed by using Prism 4 (GraphPad Software Inc., La Jolla, CA, USA, 2003).

## 4. Conclusions

This is the first study indicating the importance of the Lands’ cycle during adipocyte differentiation. By using C3H10T1/2 cells as a model, we found that LPCAT, LPEAT and LPSAT activities were enhanced during differentiation, especially with 18:2-CoA and 20:4-CoA as donors. mRNA expression of LPCAT3, an enzyme which has LPCAT, LPEAT and LPSAT activities with high substrate specificities for 18:2-CoA and 20:4-CoA, was upregulated during differentiation. Analysis of phospholipid composition of preadipocytes and adipocytes showed that there were many changes in fatty acid compositions of phospholipids, including an increase in arachidonic acid-containing species. The changes in LPLAT activities and the increase in arachidonic acid-containing phospholipid species both correlate with activities of LPCAT3, which was expressed higher in adipocytes compared to preadipocytes. This study newly suggests that phospholipid remodeling is associated with adipocyte differentiation, and that LPCAT3 might be the key enzyme for incorporating arachidonic acid into cellular membranes during differentiation of adipocytes.

## Figures and Tables

**Table 1:** LPCAT activities, LPEAT activities and LPSAT activities of preadipocytes (day 0) and adipocytes (day 8) were measured using 16:0-, 18:1-, 18:2-, 20:4- and 22:6-CoA as donors. Relative units indicate the signal intensity (area) of products, normalized by the signal intensity of internal standards. LPCAT, LPEAT and LPSAT activities increased especially with 18:2-CoA and 20:4-CoA. Three independent experiments were performed with similar results. The data represent the mean ± SD of triplicate measurements. Statistical analyses were performed with *t*-test.

Substrate	Preadipocyte (relative units)	Adipocyte (relative units)	*p* value
LPCAT activity			

16:0-CoA	7.50 ± 0.29	12.36 ± 0.68	*p* = 0.0008
18:1-CoA	3.13 ± 0.19	18.24 ± 0.61	*p* < 0.0001
18:2-CoA	198.50 ± 13.60	1821.54 ± 80.62	*p* < 0.0001
20:4-CoA	267.49 ± 11.45	1882 ± 63.12	*p* < 0.0001
22:6-CoA	0.56 ± 0.04	2.30 ± 0.18	*p* = 0.0002

LPEAT activity			

16:0-CoA	0.20 ± 0.02	0.82 ± 0.06	*p* = 0.0001
18:1-CoA	0.09 ± 0.01	3.10 ±0.17	*p* < 0.0001
18:2-CoA	1.36 ± 0.04	14.14 ± 0.63	*p* < 0.0001
20:4-CoA	2.44 ± 0.12	14.44 ± 0.40	*p* < 0.0001
22:6-CoA	0.02 ± 0.01	0.20 ± 0.02	*p* = 0.0002

LPSAT activity			

16:0-CoA	0.61 ± 0.07	1.73 ± 0.05	*p* < 0.0001
18:1-CoA	0.35 ± 0.03	1.93 ± 0.03	*p* < 0.0001
18:2-CoA	2.65 ± 0.08	17.25 ± 0.24	*p* < 0.0001
20:4-CoA	6.97 ± 0.07	28.97 ± 0.88	*p* < 0.0001
22:6-CoA	0.043 ± 0.002	0.151 ± 0.007	*p* < 0.0001

**Table 2:** Phospholipid composition of PC, PE and PS. The signal intensities for each species were summed up, and the percentage of each species was calculated. The data show the mean ± SE of three independent experiments. Statistical analyses were performed with *t*-test.

PC species	Preadipocyte (%)	Adipocyte (%)	*p* value
PC			

30:0 PC	3.13 ± 0.39	2.06 ± 0.17	ns
30:1 PC	3.67 ± 0.21	2.45 ± 0.03	0.0086**
32:0 PC	10.55 ± 0.71	5.32 ± 0.56	0.0092**
32:1 PC	9.61 ± 0.28	18.83 ± 0.75	0.0007***
32:2 PC	1.21 ± 0.17	2.95 ± 0.16	0.0037**
34:0 PC	1.98 ± 0.11	1.06 ± 0.05	0.0031**
34:1 PC	25.37 ± 1.37	22.74 ± 1.29	ns
34:2 PC	6.49 ± 0.36	7.7 ± 0.66	ns
34:3 PC	0.67 ± 0.08	1.13 ± 0.03	0.0114*
36:0 PC	0.18 ± 0.02	0.12 ± 0.01	ns
36:1 PC	6.54 ± 0.56	7.32 ± 0.47	ns
36:2 PC	12.34 ± 0.88	7.75 ± 0.17	0.0138*
36:3 PC	2.81 ± 0.12	3.51 ± 0.16	0.0452*
36:4 PC	2.09 ± 0.26	4.02 ± 0.31	0.0178*
36:5 PC	0.25 ± 0.04	0.73 ± 0.08	0.0144*
38:1 PC	1.78 ± 0.03	0.99 ± 0.03	*p* < 0.0001***
38:2 PC	2.32 ± 0.08	1.71 ± 0.12	0.0298*
38:3 PC	1.12 ± 0.06	1.22 ± 0.12	ns
38:4 PC	3.08 ± 0.17	4.36 ± 0.3	0.0395*
38:5 PC	1.75 ± 0.22	2.01 ± 0.24	ns
38:6 PC	0.35 ± 0.04	0.37 ± 0.03	ns
38:7 PC	0.04 ± 0.01	0.05 ± 0.01	ns
40:1 PC	0.22 ± 0.07	0.11 ± 0.01	ns
40:2 PC	0.27 ± 0.02	0.08 ± 0.01	0.0044**
40:3 PC	0.19 ± 0.02	0.11 ± 0.01	ns
40:4 PC	0.49 ± 0.05	0.34 ± 0.04	ns
40:5 PC	0.72 ± 0.06	0.46 ± 0.06	ns
40:6 PC	0.52 ± 0.05	0.35 ± 0.03	ns
40:7 PC	0.24 ± 0.02	0.11 ± 0.02	0.0102*

PE			

30:0 PE	0.1 ± 0.01	0.05 ± 0.01	0.0237*
32:0 PE	0.31 ± 0.04	0.24 ± 0.01	ns
32:1 PE	1.19 ± 0.12	5.74 ± 0.06	*p* < 0.0001***
32:2 PE	0.18 ± 0.02	2.66 ± 0.16	0.0002***
34:0 PE	0.32 ± 0.01	0.26 ± 0.01	0.0117*
34:1 PE	7.81 ± 0.25	9.71 ± 0.49	0.0487*
34:2 PE	2.95 ± 0.30	6.00 ± 0.23	0.0027**
34:3 PE	0.20 ± 0.02	0.93 ± 0.07	0.001***
36:1 PE	14.01 ± 0.06	7.02 ± 0.40	0.0001***
36:2 PE	9.93 ± 0.69	7 ± 0.03	0.0254*
36:3 PE	1.05 ± 0.10	1.32 ± 0.02	ns
36:4 PE	2.80 ± 0.11	4.78 ± 0.14	0.0009***
36:5 PE	0.34 ± 0.02	1.64 ± 0.08	0.0002***
38:1 PE	0.69 ± 0.04	0.21 ± 0.02	0.0009***
38:2 PE	1.55 ± 0.24	1.09 ± 0.07	ns
38:3 PE	5.09 ± 0.54	5.28 ± 0.02	ns
38:4 PE	30.45 ± 1.32	29.95 ± 0.17	ns
38:5 PE	5.32 ± 0.16	5.13 ± 0.20	ns
38:6 PE	0.7 ± 0.06	1.05 ± 0.09	ns
38:7 PE	0.06 ± 0.01	0.29 ± 0.02	0.0006***
40:2 PE	0.81 ± 0.06	0.24 ± 0.03	0.0021**
40:3 PE	1.14 ± 0.04	0.89 ± 0.15	ns
40:4 PE	6.49 ± 0.49	2.88 ± 0.19	0.0048**
40:5 PE	1.62 ± 0.10	1.37 ± 0.04	ns
40:6 PE	3.53 ± 0.15	3.11 ± 0.06	ns
40:7 PE	0.69 ± 0.16	0.74 ± 0.05	ns
42:9 PE	0.38 ± 0.03	0.28 ± 0.01	0.0491*
42:10 PE	0.08 ± 0.01	0.08 ± 0.01	ns

**PS**			

34:0 PS	0.67 ± 0.16	0.37 ± 0.05	ns
34:1 PS	3.76 ± 1.87	5.65 ± 3.54	ns
34:2 PS	0.79 ± 0.01	0.83 ± 0.1	ns
36:1 PS	37.45 ± 1.23	34.94 ± 1.87	ns
36:2 PS	6.82 ± 0.40	5.8 ± 0.4	ns
36:4 PS	1.01 ± 0.04	1.27 ± 0.09	ns
38:1 PS	2.37 ± 0.16	1.86 ± 0.08	ns
38:2 PS	1.85 ± 0.26	1.77 ± 0.21	ns
38:3 PS	6.64 ± 0.40	7.8 ± 0.38	ns
38:4 PS	12.17 ± 0.96	14.7 ± 1.12	ns
38:5 PS	1.30 ± 0.15	1.14 ± 0.08	ns
40:1 PS	1.54 ± 0.10	1.11 ± 0.10	ns
40:2 PS	1.28 ± 0.14	1.41 ± 0.16	ns
40:3 PS	1.61 ± 0.32	2.95 ± 0.42	ns
40:4 PS	9.86 ± 0.91	8.41 ± 0.36	ns
40:5 PS	4.00 ± 0.80	4.05 ± 0.71	ns
40:6 PS	4.27 ± 0.16	3.8 ± 0.15	ns
40:7 PS	0.23 ± 0.02	0.19 ± 0.01	ns
42:5 PS	0.77 ± 0.06	0.48 ± 0.07	ns
42:7 PS	0.64 ± 0.07	0.49 ± 0.06	ns
42:8 PS	0.54 ± 0.08	0.59 ± 0.05	ns
42:9 PS	0.42 ± 0.02	0.39 ± 0.04	ns

ns, not significant;

* *p* < 0.05;

** *p* < 0.01;

*** *p* < 0.001.

**Table 3:** Fatty acid composition of PC and PE. The signal intensities for each species were summed up, and the percentage of each species was calculated. The data show the mean of two independent experiments.

PC species	Preadipocyte (%)	Adipocyte (%)
PC		

16:0/16:0 PC	12.78	8.97
16:0/16:1 PC	4.13	7.45
16:0/18:0 PC	13.16	12.58
16:0/18:1 PC	24.17	20.64
16:0/18:2 PC	3.58	4.32
16:0/18:3 PC	2.51	3.33
16:0/20:4 PC	1.12	2.11
16:0/22:6 PC	0.43	0.42
18:0/18:1 PC	4.57	4.73
18:0/18:2 PC	2.61	4.21
18:0/18:3 PC	6.63	7.31
18:0/20:4 PC	1.37	2.61
18:0/22:6 PC	2.06	1.71
18:1/18:1 PC	7.90	5.51
18:1/18:2 PC	10.91	11.24
18:1/18:3 PC	1.66	2.28
18:1/20:4 PC	0.42	0.60
18:1/22:6 PC	12.78	8.97

PE		

16:0/16:0 PE	0.30	0.22
16:0/16:1 PE	0.93	6.12
16:0/18:0 PE	7.08	3.63
16:0/18:1 PE	6.96	6.05
16:0/18:2 PE	0.38	0.85
16:0/18:3 PE	0.05	0.17
16:0/20:4 PE	2.00	4.16
16:0/22:6 PE	0.24	0.43
18:0/18:1 PE	26.75	19.09
18:0/18:2 PE	2.83	3.41
18:0/18:3 PE	0.37	0.54
18:0/20:4 PE	34.77	40.20
18:0/22:6 PE	1.22	1.18
18:1/18:1 PE	10.21	7.83
18:1/18:2 PE	0.94	0.91
18:1/18:3 PE	0.12	0.13
18:1/20:4 PE	3.41	3.72
18:1/22:6 PE	1.44	1.36

**Table 4:** Primers used for quantitative PCR analysis.

Primers	Sequence
LPCAT1 forward	GTGCACGAGCTGCGACT
LPCAT1 reverse	GCTGCTCTGGCTCCTTATCA
LPCAT2 forward	GTCCAGCAGACTACGATCAGTG
LPCAT2 reverse	CTTATTGGATGGGTCAGCTTTTC
LPCAT3 forward	TCAGGATACCTGATTTGCTTCCA
LPCAT3 reverse	GGATGGTCTGTTGCACCAAGTAG
LPCAT4 forward	TTCGGTTTCAGAGGATACGACAA
LPCAT4 reverse	AATGTCTGGATTGTCGGACTGAA
LPEAT1 forward	CTGAAATGTGTGTGCTATGAGCG
LPEAT1 reverse	TGGAAGAGAGGAAGTGGTGTCTG
PPARγ2 forward	TATGCTGTTATGGGTGAAACTCTGG
PPARγ2 reverse	GTCAAAGGAATGCGAGTGGTCT
36B4 forward	CTGAGATTCGGGATATGCTGTTG
36B4 reverse	AAAGCCTGGAAGAAGGAGGTCTT
